# Accelerated long-term forgetting: A sensitive paradigm for detecting subtle cognitive impairment and evaluating BACE1 inhibitor efficacy in preclinical Alzheimer's disease

**DOI:** 10.3389/frdem.2023.1161875

**Published:** 2023-04-18

**Authors:** Masuo Ohno

**Affiliations:** Center for Dementia Research, Nathan Kline Institute, Orangeburg, NY, United States

**Keywords:** accelerated long-term forgetting, Alzheimer's disease, preclinical stage, BACE1 inhibitor, amyloid-β (Aβ), mouse model, clinical trials

## Abstract

Given a long preclinical stage of Alzheimer's disease (AD) continuum before the onset of dementia, there is a growing demand for tools capable of detecting the earliest feature of subtle cognitive impairment and optimizing recruitment to clinical trials for potentially disease-modifying therapeutic interventions such as BACE1 inhibitors. Now that all BACE1 inhibitor programs in symptomatic and prodromal AD populations have ended in failure, trials need to shift to target the earlier preclinical stage. However, evaluating cognitive efficacy (if any) in asymptomatic AD individuals is a great challenge. In this context, accelerated long-term forgetting (ALF) is emerging as a sensitive cognitive measure that can discriminate between presymptomatic individuals with high risks for developing AD and healthy controls. ALF is characterized by increased forgetting rates over extended delays (e.g., days, weeks, months) despite normal learning and short-term retention on standard memory assessments that typically use around 30-min delays. This review provides an overview of recent progress in animal model and clinical studies on this topic, focusing on the utility and underlying mechanism of ALF that may be applicable to earlier diagnosis and BACE1 inhibitor efficacy evaluation at a preclinical stage of AD.

## Introduction

**Figure 1:**
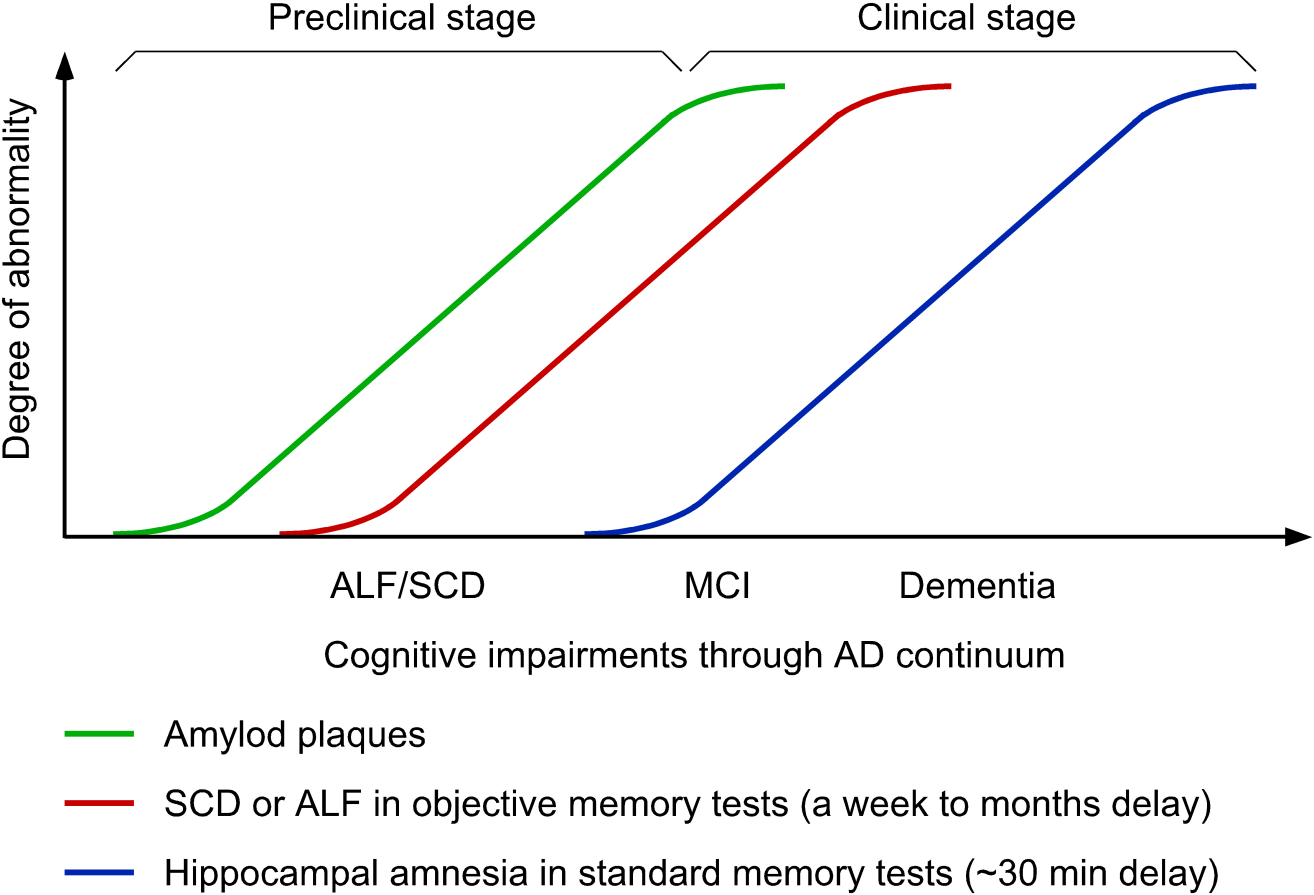
Time-course changes in cognitive symptoms along Alzheimer's disease (AD) continuum. ALF, accelerated long-term forgetting; SCD, subjective cognitive decline; MCI, mild cognitive impairment.

The β-secretase BACE1, which initiates amyloid-β (Aβ) production, is a long-standing prime therapeutic target for Alzheimer's disease (AD) based on solid evidence that Aβ increase is the first event driving subsequent pathological changes and cognitive symptoms (Hanseeuw et al., 2019; Jack et al., 2019). However, BACE1 inhibitors tested to date in clinical trials have yielded no benefit first in patients with mild-to-moderate AD and more recently in early or prodromal AD populations (Imbimbo and Watling, 2019; McDade et al., 2021; Bazzari and Bazzari, 2022). Given Aβ deposition commencing decades before the symptom onset (Bateman et al., 2012) (Figure 1), symptomatic AD brains already harbor significant Aβ burden less sensitive to BACE1 inhibitor interventions (Peters et al., 2018) and even if reduced, downstream detrimental consequences of Aβ may continue. Prior clinical trials have targeted Aβ reduction at least by ~50% and in many cases by more than 70%, resulting in discontinuation due to futility or toxicity issues (Imbimbo and Watling, 2019; McDade et al., 2021; Bazzari and Bazzari, 2022). In particular, cognitive worsening (rather than expected improvement) was found using the highest dosage of multiple BACE1 inhibitors. This is most likely reflective of side effects of overdosed BACE1 inhibitor drugs given that the Icelandic mutation (A673T) in the *amyloid-*β *precursor protein (APP)* gene resulting in only ~30% lifelong reduction of Aβ is protective against AD and age-related cognitive decline (Jonsson et al., 2012; Martiskainen et al., 2017). Moreover, given that the inhibitor drugs tested have limited or no selectivity for BACE1 over the isoform BACE2, we cannot completely rule out the possibility that BACE2 inhibition may contribute to cognitive worsening (Hampel et al., 2021; McDade et al., 2021). Collectively, low-dose BACE1-specific inhibitor trials initiated at the earliest preclinical stage of AD is most promising, while evaluating the cognitive efficacy (if any) in asymptomatic individuals at a risk of developing AD is challenging (Mortamais et al., 2017).

Accelerated long-term forgetting (ALF) refers to faster forgetting of episodic memories over prolonged periods (days, weeks, months) despite normal acquisition and short-term retention ranging from 20 to 40 min, which is typically used in clinical practice of objective memory testing (Elliott et al., 2014; Geurts et al., 2015). In animal models of AD, ALF proves to represent a sensitive measure to successfully detect subtle cognitive phenotypes in young 5XFAD and PDAPP transgenic mice that retain intact hippocampal long-term potentiation (LTP: a synaptic plasticity model for episodic memory formation) and the ability to learn contextual and spatial memory tasks (presymptomatic AD models) (Kimura and Ohno, 2009; Beglopoulos et al., 2016; Ohno, 2021). Consistent with these findings, emerging clinical investigations reveal that ALF during longer delays (1 week to 6 months) is one of the earliest cognitive changes that occur at an asymptomatic stage in ApoE ε4 carriers (Zimmermann and Butler, 2018; Tort-Merino et al., 2021a) and individuals with familial AD (FAD) (Weston et al., 2018; O'Connor et al., 2020; Yang et al., 2021) or subjective cognitive decline (SCD) (Manes et al., 2008; Tort-Merino et al., 2021b), who are still normal in standard or short-term memory tests (Figure 1). This article reviews recent advances in this field, which demonstrate (1) distinct functions and mechanisms underlying ALF and classic hippocampal amnesia in AD continuum and (2) the utility of ALF and its potential biomarkers in optimal design of next-generation BACE1 inhibitor trials at preclinical AD stages.

## ALF and the underlying mechanisms in preclinical AD

The term ALF was previously designated as long-term amnesia (Kapur et al., 1997; Mayes et al., 2003) that describes a phenomenon that episodic memories are normally learned and retained for standard delays around 30 min, whereas considerable impairments are observed if the same tests are given at extended delays ranging from days to months. This phenomenon was first described and has been extensively studied in patients with epilepsy (Blake et al., 2000; Mameniškiene et al., 2020). Recently, ALF has been gaining a great deal of attention as a sensitive measure for detecting subtle memory dysfunction in other neurological conditions, including traumatic brain injury (Lah et al., 2017), stroke (Geurts et al., 2019; Lammers et al., 2022), limbic encephalitics (Helmstaedter et al., 2019) and enhanced risks for AD (Manes et al., 2008; Weston et al., 2018; Zimmermann and Butler, 2018; O'Connor et al., 2020; Tort-Merino et al., 2021a,b; Yang et al., 2021). ALF associated with neurodegenerative diseases can be considered as a harbinger of preclinical dementia and allows the earliest diagnostic detection of AD way before the onset of clinically measurable cognitive deficits in standard tests that underestimate subtle cognitive changes.

Does ALF that becomes evident only after extended delays reflect neurobiological mechanisms that are qualitatively distinct from those underlying typical forgetting in classical hippocampal amnesia where memories decay faster within a short time window (generally ~30 min) after learning? (Mayes et al., 2019). Otherwise, two different types of memory disorders after short- and long-term delays may represent only quantitatively different expression of the same underlying mechanisms (Cassel and Kopelman, 2019). Learning rapidly triggers local changes in activated hippocampal synapses and episodic memory formation initially requires synaptic plasticity (e.g., LTP) and structural changes within hippocampal circuits. As memories mature with time, they increasingly become independent of the hippocampus and memory traces are gradually stabilized and eventually consolidated into remote memories within cortical networks, especially medial prefrontal cortical regions including the anterior cingulate cortex (ACC) (systems consolidation) (Frankland and Bontempi, 2005; Tonegawa et al., 2018; Klinzing et al., 2019). There is currently no consensus about the exact time delay after which ALF occurs or whether ALF falls into the time frame of deficient systems consolidation processing. Importantly, evidence from mouse model studies is accumulating to support the hypothesis that ALF following intact hippocampal memory encoding may reflect the impairment of systems memory consolidation in preclinical AD.

**Figure 2:**
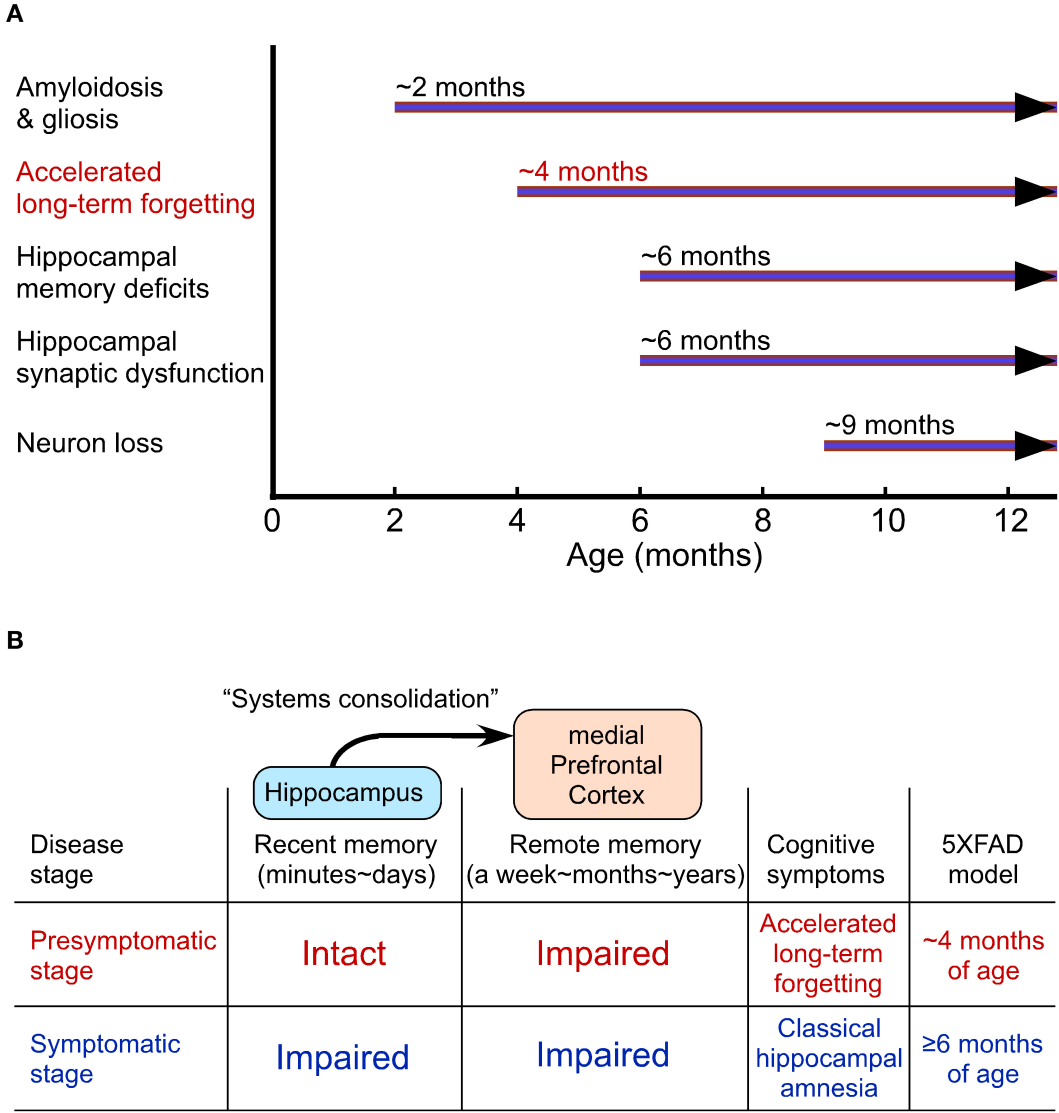
Accelerated long-term forgetting (ALF) as one of the earliest cognitive changes that can be identified in a preclinical stage of Alzheimer's disease (AD). **(A)** The onset of AD-like traits in 5XFAD model mice. **(B)** Cortical dysfunction-related ALF over extended delays precedes the onset of classical hippocampal amnesia on standard memory tests with short delays.

ALF is well characterized in the 5XFAD and PDAPP mouse models of AD (Kimura and Ohno, 2009; Beglopoulos et al., 2016; Ohno, 2021). 5XFAD mice represent one of the earliest-onset and most aggressive amyloid models based on the overexpression of human APP and presenilin 1 (PS1) harboring five FAD mutations (Oakley et al., 2006; Ohno et al., 2006), providing a presymptomatic AD model at the young age (Figure 2A). 5XFAD mice develop Aβ deposition as early as ~2 months of age, exhibiting significant memory impairments on standard hippocampus-dependent paradigms (e.g., contextual fear conditioning, Morris water maze) at ~6 months concomitant with moderate Aβ accumulation and the onset of Schaffer collateral-CA1 synaptic dysfunctions (basal transmission and LTP) (Oakley et al., 2006; Ohno et al., 2006; Kimura and Ohno, 2009; Ohno, 2009; Devi and Ohno, 2010, 2015; Kimura et al., 2010). Faithfully recapitulating a time lag between the onset of Aβ build-up in human AD brain and that of objective memory impairments on standardized cognitive tests (Bateman et al., 2012), this model has an asymptomatic phase (2–6 months of age) on hippocampal learning tasks (Figure 2A), during which Aβ continues to increase dramatically. Remarkably, while the standard procedure of 24-h memory assessment is not sensitive enough for detecting subtle impairment in the contextual fear conditioning in 5XFAD mice at this stage (4 months), ALF is evident when a longer delay (30 days) intervenes between training and memory testing (Kimura and Ohno, 2009). Furthermore, a recent study reports that 5XFAD mice at ~2 months of age already show significantly impaired LTP in the prefrontal cortex concomitant with its considerable Aβ deposition (Chen et al., 2022), preceding the onset of hippocampal LTP deficits (Kimura and Ohno, 2009). Similarly, pre-pathological PDAPP mice are also normal in hippocampus-dependent acquisition and spatial memory performance tested 10 min after water maze training, whereas they show ALF after a long delay (7 days) (Beglopoulos et al., 2016). Notably, young PDAPP mice exhibit impairments in 7-day memory retrieval-associated glucose uptake (not in basal uptake levels) in cortical areas rather than in the hippocampus. Together, AD mouse models at a very incipient stage can perform normally in memory tests after a short delay reminiscent of negative diagnosis of classical hippocampal amnesia on standard memory tests in asymptomatic AD individuals (Figure 2B). After a prolonged delay, ALF emerges concomitant with cortical dysfunction in presymptomatic mouse models of AD, strongly suggesting that ALF may reflect impaired remote memory consolidation in the medial prefrontal cortex.

Recent clinical investigations show that ALF over an extended retention interval is detectable in presymptomatic individuals with high genetic risks for AD. First, Weston et al. (2018) reported that asymptomatic carriers of autosomal dominant AD mutations, who were on average 7 years from estimated symptom onset, were normal in initial learning and 30-min memory recall on three tasks (word list, short story, and complicated visual figure) but showed ALF as compared with non-carrier controls from the same families when they were tested 7 days later. A correlation between ALF and SCD was found together with the increased severity of ALF with proximity to the symptom onset. Similarly, verbal and visual measures of ALF (7-day delay) were detected in clinically normal individuals who carried *APP/PS1* FAD mutations but remained intact in the standard 30-min memory performance, starting approximately a decade prior to estimated symptom onset (O'Connor et al., 2020). More recently, Yang et al. (2021) identified a couple of senescence-related blood borne factors whose changes were closely associated with ALF after 7-day delay, independent of age, in asymptomatic individuals with FAD mutations. The study demonstrated a possibility for promising diagnostic biomarkers for the prediction of ALF at a preclinical stage of AD. It has also been studied whether ALF may occur in asymptomatic individuals who carry the principal genetic risk factor for developing a sporadic form of AD. *Apolipoprotein E (APOE)* ε4 status is associated with ALF over 7-day delay in healthy people who have no discernible change in memory encoding and forgetting over the first 30 min (Zimmermann and Butler, 2018). Notably, the severity of ALF increases linearly with the number of copies of the *APOE* ε4 allele. Furthermore, Tort-Merino et al. (2021a) found ALF over 3 months in asymptomatic *APOE* ε4 carriers and a significant negative correlation between the forgetting rate and the CSF Aβ42/p-tau ratio, providing evidence that ALF may serve as cognitive predisposition toward developing AD.

It is important to note that ALF is often observed in parallel with SCD in presymptomatic carriers of FAD mutations and *APOE* ε4 allele (Weston et al., 2018; O'Connor et al., 2020; Tort-Merino et al., 2021a) (Figure 1). The key features of SCD include (1) the self-experienced persistent decline of cognitive function compared with previous normal levels that is unrelated to acute events and (2) the normal performance on standard cognitive tests used for classifying mild cognitive impairment (MCI) (Jessen et al., 2014). Meanwhile, MCI is a clinical condition that is not only characterized by subjective cognitive complaints but also confirmed by poor performance on standard memory assessments. MCI subjects who reach learning criteria equivalent to healthy controls with additional trials exhibit an increased rate of forgetting within 30-min delay and more importantly a greater rate of forgetting is evident at 1-week delay (i.e., ALF) (Walsh et al., 2014) (Figure 1). However, it seems difficult to reveal increased forgetting rates over longer delays in MCI patients compared with age-matched controls without controlling them for equated initial learning (Grönholm-Nyman et al., 2010; Alber et al., 2014; Vallet et al., 2016). In comparing between SCD and MCI subjects, ALF at 6-week delay is detectable in both groups, while standard 30-min memory testing is sensitive enough to detect impairments only in the MCI group (Manes et al., 2008). A recent study also demonstrates ALF over 3 months in cognitively unimpaired individuals with high SCD ratings, particularly, in those with abnormal Aβ42 levels (Tort-Merino et al., 2021b). Given that SCD assessment may be affected by many artifact factors including the way of quantifying cognitive complaints, recruitment settings and the threshold used for cognitive normality, ALF provides a more powerful preclinical cognitive measure that can detect objectively subtle changes at the earliest stage of AD continuum. It is tempting to speculate that ALF detectable in SCD individuals before diagnosis may be associated with altered functional connectivity in prefrontal cortical areas including the ACC (Yuan et al., 2022), which shows highest regional Aβ load associated with local atrophy by PET imaging (Chételat et al., 2010). The findings support the concept that changes in different brain structures may underlie two distinct forms of amnesia, classical hippocampal amnesia (MCI and thereafter) and ALF at an earlier preclinical stage reflecting impaired cortical long-term memory consolidation. Further investigation of neuroimaging biomarkers and neural network characterization is required to finely locate key brain regions responsible for ALF in preclinical AD.

## ALF and potential biomarkers to evaluate BACE1 inhibitors in preclinical AD

Although ALF is barely used as a preclinical cognitive marker to evaluate therapeutic interventions, a recent animal model study demonstrated that ALF in 4-month-old 5XFAD mice can be rescued by the selective BACE1 inhibitor GRL-8234 (39-fold selectivity vs. BACE2) (Chang et al., 2011) administered during a long delay (28 days) after training in the contextual fear conditioning (Ohno, 2021). The standard 24-h memory assessment was not sensitive enough for detecting subtle impairment at this stage. These findings provide an experimental foundation for the utility of ALF as an early feature of subtle cognitive impairment that is applicable to presymptomatic efficacy evaluation of BACE1 inhibitors. Interestingly, 5XFAD mice exhibit age-dependent increases in serum Aβ42 concentrations up to 4.5 months that correlate with Aβ accumulation in the brain, while decreased serum Aβ42 coincides with the subsequent development of widespread and large plaques (Botella Lucena et al., 2022). It is possible that an initial increase of serum Aβ42 may represent a biomarker that correlates with the occurrence of ALF at a preclinical AD phase and is useful for preventive BACE1 inhibitor evaluation. Meanwhile, unlike GRL-8234, other BACE1 inhibitor drugs tested to date have poor or no selectivity for BACE1 over its homolog BACE2 (up to ~3 folds) and their clinical trials at symptomatic AD stages were halted because of futility or adverse effects including cognitive worsening at the highest dosage (Imbimbo and Watling, 2019; McDade et al., 2021; Bazzari and Bazzari, 2022). Given recent evidence for the physiological role of BACE2 as an AD-suppressor gene (Alić et al., 2021; Luo et al., 2022), further investigation is required to address whether cross-inhibition of BACE2 activity by non-selective or partially selective BACE1 inhibitors may diminish the benefit of BACE1 inhibition or contribute to the untoward worsening effect on ALF (if any) in preclinical AD.

What mechanisms may underlie the occurrence of ALF and its prevention with BACE1 inhibitors? Among senescence-related blood borne factors that directly affect neurogenesis and synaptic plasticity (Villeda et al., 2011; Katsimpardi et al., 2014; Gan and Südhof, 2019), downregulation of the rejuvenating factor thrombospodin-4 (THBS4) and upregulation of the pro-aging factor CC chemokine ligand 11 (CCL11) or growth differentiation factor 11 (GDF11) in plasma correlate with ALF after 7-day delay in presymptomatic FAD mutation carriers who are normal on standard 30-min memory testing (Yang et al., 2021). The findings indicate that these blood borne factors may serve as potential biomarkers for ALF, although the underlying mechanisms and responsiveness to BACE1 inhibitors remain to be determined.

While divergent mechanisms are proposed to account for forgetting (Davis and Zhong, 2017; Ryan and Frankland, 2022), Rac1, a small GTPase, plays a key role not only in natural forgetting in health but also in pathological forgetting in disease. Interestingly, Rac1 is aberrantly activated by exposure to Aβ (Manterola et al., 2013) in the brains of young AD model mice including 3-month-old APP/PS1 and 6-week-old 3xTg-AD as well as in brain and plasma samples of AD patients (Borin et al., 2018; Wu et al., 2019). Moreover, Rac1 inhibition rescues faster forgetting in young APP/PS1 mice that retains intact spatial learning ability and memory up to 4 h after training in the Morris water maze (Wu et al., 2019). These results suggest that excessively activated Rac1-mediated pathways may contribute to ALF in preclinical AD.

Endocytosis of AMPA receptors containing the GluA2 subunit from the postsynaptic membrane is known to mediate forgetting by weakening synaptic connectivity among memory engram cells (Davis and Zhong, 2017; Ryan and Frankland, 2022). This mechanism is also involved in physiological forgetting of long-term memory (Dong et al., 2015; Migues et al., 2016) and Aβ-induced synaptic depression and dendritic spine loss (Hsieh et al., 2006). Notably, blocking GluA2-containing AMPA receptor endocytosis in 1.5-month-old APP23/PS45 mice after a single inhibitory avoidance training prevents the subsequent long-term forgetting over 30 days (Dong et al., 2015). In addition to Aβ-mediated mechanisms, β-secretase-cleaved C-terminal fragment (β-CTF or C99), an intermittent β-metabolite of APP, may also contribute to faster forgetting in preclinical AD, *via* well-defined endosomal enlargement that precedes Aβ accumulation and accompanies accelerated endocytosis (Nixon, 2017). β-CTF-dependent (but Aβ-independent) overactivation of Rab5, a small GTPase associated with early endosome, has been shown to cause endosomal abnormalities in neurons from AD patients (Kim et al., 2016), induced pluripotent stem cells (iPSCs) derived from AD patients (Israel et al., 2012) and CRISPR/Cas9-generated iPSC lines carrying FAD mutations (Kwart et al., 2019). Remarkably, a recent study demonstrates that directly overactivating Rab5 in mice recapitulates many key features of early AD including enlarged endosome pathology and accelerated endocytosis of GluA2-containing AMPA receptors (Pensalfini et al., 2020). Given that both Aβ and β-CTF are responsive to reductions by BACE1 inhibitors that rescues ALF in young 5XFAD mice (Devi et al., 2015; Ohno, 2021), it is important to explore signaling mechanisms underlying aberrant AMPA receptor removal associated with ALF. Further mechanistic understanding of ALF and validated biomarkers will increase the utility of ALF as a new standard cognitive measure for earlier diagnosis and BACE1 inhibitor evaluation in preclinical AD populations.

## Discussion

Whereas this review is focused on BACE1 inhibitors whose efficacy in rescuing ALF has been demonstrated in asymptomatic AD mouse models (Ohno, 2021), ALF assessment is expected to provide a great opportunity to sensitively evaluate other Aβ-reducing interventions such as γ-secretase modulators in a long preclinical phase of AD continuum. In particular, ALF should be more powerful in testing preventive Aβ-lowering therapy, if applied in combination with the use of earliest biomarkers such as increased blood Aβ42 concentrations indicative of the initiation of Aβ accumulation in the brain (Botella Lucena et al., 2022).

Now that all BACE1 inhibitor programs in symptomatic and prodromal AD have ended in failure (Imbimbo and Watling, 2019; McDade et al., 2021; Bazzari and Bazzari, 2022), trials need to shift to target the earlier preclinical stage across the AD spectrum such as secondary prevention (presymptomatic populations) and primary prevention [before Aβ build-up preceding symptom onset by ~15 years (Bateman et al., 2012)] (Figure 1). As a reliable readout of subtle cognitive impairment that precedes symptom onset in standard memory tests, ALF is useful for objectively detecting cognitive benefits (if any) that may be produced by lower, physiologically relevant levels of BACE1 inhibition (~30% or even smaller). This idea is supported by clinical observations that the Icelandic APP mutation (A673T) that reduces Aβ by 28% is protective against AD and age-related cognitive decline (Jonsson et al., 2012; Martiskainen et al., 2017). While ALF represents an easy-to-test paradigm with additional delayed test(s) for memory recall (ranging from a week to several months), the standardized procedure (e.g., delay intervals, test materials) is instrumental in establishing ALF as an objective measure for cognitive decline in preclinical AD populations (Rami et al., 2023).

The major concern over prior clinical trials is that high-dose BACE1 inhibitor strategies tested to date, which often achieved >70% Aβ reduction, suffered from the side effects, especially, unexpected cognitive worsening at the highest dosing of most BACE1 inhibitors in prodromal AD (Imbimbo and Watling, 2019; McDade et al., 2021; Bazzari and Bazzari, 2022). In this regard, it is important to note that a growing number of BACE1 substrates besides APP uncover new physiological roles of this protease (Barao et al., 2016; Hampel et al., 2021). Although further research is needed, cognitive worsening, which occurs soon after treatment and is non-progressive and reversible after withdrawal of high-dose BACE1 inhibitors, may be associated with synaptic BACE1 substrates such as seizure protein 6 (SEZ6) involved in maintaining spine dynamics (Filser et al., 2015; Blume et al., 2018; Zhu et al., 2018a,b), close homolog of L1 (CHL1) related to axonal organization in adulthood (Ou-Yang et al., 2018; Vassar, 2019), and so forth. In fact, BACE1 knockouts and BACE1 inhibitors administered at an overdose level toxic to normal adult mice induce synaptic and/or cognitive adverse effects at least in part through these detrimental mechanisms. Importantly, partial BACE1^+/−^ reduction and lower-dose BACE1 inhibitors are, however, devoid of such mechanism-based side effects (Ohno, 2016; Zhu et al., 2018b), supporting the promise of a rational, low-dose approach initiated at the earliest preclinical stage of AD. As a proof of concept, it was recently demonstrated that chronic administration of the selective BACE1 inhibitor GRL-8234 at the safe dosage within ~50% β-cleavage suppression (Devi et al., 2015) can rescue ALF over an extended delay in 5XFAD mice at an asymptomatic stage that retain normal performance on the standard memory paradigm with a short delay (Ohno, 2021).

One of the weaknesses of current clinical trials is that determining the efficacy/safety of BACE1 inhibitors largely depends on final cognitive outputs (i.e., lack of biomarker-based end points). Further study is required to establish blood-based biomarkers such as Aβ42 elevations (Botella Lucena et al., 2022) and Aβ PET imaging analysis in defined brain structures (Syvänen et al., 2022) that are closely associated with the occurrence of ALF and its rescue with BACE1 inhibitors. Undoubtedly, more work also needs to be done to better understand the mechanisms of cognitive worsening and identify the underlying substrates that can serve as markers to track side effects of overdosed BACE1 inhibitors. In this context, given hormetic roles of Aβ peptides in cognitive and synaptic functions, it should be kept in mind that BACE1 inhibitors should be given in a way that would return excess Aβ levels toward normal but certainly not to below-physiological levels especially when they are applied at time well before clinical symptoms (Ohno et al., 2004; Ohno, 2016). Collectively, optimal clinical trials should be designed by initiating interventions with selective BACE1 inhibitors at the earliest preclinical AD stage, utilizing very sensitive cognitive paradigms and relevant biomarkers for efficacy, and targeting safe levels of reduction in β-secretase activity (~50% or lower) supported by safety biomarker assessments to avoid potential side effects. This can be dealt with successfully if cognitively normal individuals on standard memory testing are reliably diagnosed as at-risk preclinical AD suitable for preventive BACE1 inhibitor interventions according to genetic predisposition, ALF assessment and related biomarker profiles. Advances in this line of research are highly expected to form the basis of personalized medicine for AD.

## Author contributions

The author confirms being the sole contributor of this work and has approved it for publication.
